# Comparison of erector spinae fatigability between female patients with Parkinson’s disease and healthy individuals: a cross sectional pilot study

**DOI:** 10.1186/s12883-022-02719-w

**Published:** 2022-05-23

**Authors:** Yukihide Nishimura, Hiroyuki Tsuboi, Ken-Ya Murata, Yuta Minoshima, Hideyuki Sato, Yuichi Umezu, Fumihiro Tajima

**Affiliations:** 1 Department of Rehabilitation Medicine, Iwate Medical University, Yahaba-cho Shiwa-gun, Iwate, 028-3695 Japan; 2 Rehabilitation Division, Iwate Medical University Hospital, Iwate, Japan; 3 Department of Neurology, Wakayama Medical University, Wakayama, Japan; 4 Division of Rehabilitation, Wakayama Medical University Hospital, Wakayama, Japan; 5 Department of Rehabilitation, Konan Medical Center, Hyogo, Japan; 6 Department of Rehabilitation, Kokura Rehabilitation Hospital, Fukuoka, Japan; 7 Department of Rehabilitation Medicine, Wakayama Medical University, Wakayama, Japan

**Keywords:** Parkinson’s disease, Paraspinal muscles, Electromyography, Muscle Fiber type, Rehabilitation

## Abstract

**Background:**

Postural abnormality is one of the main symptoms of Parkinson’s disease (PD). The erector spinae muscles play an important role in maintaining an upright posture, but the fatigability of the erector spinae in patients with PD is unknown. The purpose of this study was to compare the trunk extension maximum voluntary contraction (MVC) and the fatigability of the erector spinae between female patients with PD and healthy volunteers.

**Methods:**

Th participants of this cross-sectional pilot study comprised 19 patients with PD and nine healthy volunteers matched for sex, age, and physical characteristics as a control group. The MVC of all participants was measured, and after sufficient rest, the Sørensen back endurance test was conducted to the point of exhaustion. The muscle activity of the erector spinae during the Sørensen back endurance test was measured using surface electromyography. The median frequency (MF) slope, which is an index of fatigability, was calculated from the recorded surface muscle activity by means of power spectrum analysis using a Fast Fourier transformation.

**Results:**

Nine of the 19 patients with PD were unable to perform the Sørensen back endurance test, and a lower proportion of the PD group were able to perform it compared with the control group. The MVC of those patients with PD who were able to perform the Sørensen back endurance test was lower than that of the control group, and the time for which the pose could be maintained was shorter. There was no significant difference between the MF slope on the left and right side in the PD group, and it was higher on both sides than in the control group.

**Conclusion:**

This is the first study to demonstrate a reduction of maximum muscle strength and great fatigability of the erector spinae in patients with PD. This discovery strongly underlines the need for paraspinal muscle training from an early stage with the aim of preventing the progression of postural abnormality in patients with PD.

## Background

Postural abnormality is one of the main symptoms of Parkinson’s disease (PD). This most commonly takes the form of trunk anteflexion, but scoliosis is also seen. The erector spinae muscles are essential to trunk movement and the maintenance of an upright posture, and weakness of these muscles exacerbates postural abnormality [1]. The erector spinae are controlled by the corticospinal and reticulospinal tracts [2]. Because patients with PD develop extrapyramidal symptoms, these may affect the erector spinae at an early stage soon after onset.

A previous magnetic resonance imaging (MRI) study of the paraspinal muscle mass of patients with PD reported that they were asymmetrical, with greater muscle atrophy on the side on which the PD symptoms first became evident than on the opposite side in eight of 10 patients [3]. In another study, the proportion of type 1 fibers in lateral vastus muscle biopsies from patients with PD was higher than those from either young or older healthy individuals [4]. Skeletal muscle fatigability also contributes to PD symptoms, and a number of studies have suggested that mitochondrial dysfunction may be involved [5, 6]. Thus, there is no association between the proportion of muscle fiber types and mitochondrial function in patients with PD. To our knowledge, no study has yet addressed the fatigability of the erector spinae in patients with PD. Evaluating the fatigability of the erector spinae in patients with PD might contribute to the development of effective interventions for preventing the progression of postural abnormality or even improving it, and is therefore of clinical value.

One method of evaluating the fatigability of the erector spinae is to use electromyography (EMG) to measure muscle activity during the Sørensen back endurance test [7, 8], in which the subject’s lower extremities are fixed to a bed and they maintain their trunk in the horizontal position without support. The median frequency (MF) calculated by frequency power spectrum analysis of the recorded surface muscle activity attenuates over time. Because the degree of MF attenuation is highly reliable [9] and is associated with the proportions of muscle fiber types [10], it is widely used as an indicator of erector spinae fatigability by clinicians and researchers [11–15].

In a systematic literature review aimed at examining the validity and applicability of methods for the assessment of erector spinae muscle fatigability in everyday clinical rehabilitation practice, the Sørensen back endurance test combined with surface electromyography spectral analysis has been shown to be the most widely used and the comparatively most optimal modality currently available to assess objective erector spinae muscle fatigability. However, patients who have severe erector spinae muscle weakness might not be able to maintain the pose for even just a few seconds [16]. Therefore, whether patients with PD with abnormal posture are capable of performing the Sørensen back endurance test is unknown.

Against this backdrop, we hypothesized that maximum muscle strength is severely diminished and fatigability of the erector spinae is high from an early stage in patients with PD. The objectives of this pilot study were (1) to verify whether the Sørensen back endurance test can be used to evaluate muscle fatigability of the erector spinae in patients with PD, and (2) to measure the fatigability of the erector spinae on the side on which PD symptoms first appeared and the opposite side in patients with PD, to compare the results with data on the erector spinae of healthy volunteers.

## Methods

### Study design

This was a cross-sectional pilot study that was conducted following our previously reported measurement protocol.^13,14,)^ This study was approved by the Research Ethics Committee of Wakayama Medical University (approval number: 1307). All methods were carried out in accordance with relevant guidelines and regulations. Written informed consent was obtained in advance from all participants.

### Participants

Nineteen patients diagnosed with PD and undergoing outpatient treatment at the Department of Neurology of Wakayama Medical University Hospital took part in this study. The cross-sectional area of the erector spinae is larger in elderly males than in elderly females [17], and the prevalence of PD in Japan [18] and Korea [19]^.^ is higher in females overall. The inclusion criteria thus were: age ≥ 65, female sex, and modified Hoehn & Yahr (H-Y) Stage < 3 PD. The exclusion criteria were: (1) spinal anteflexion of ≥45° (camptocormia) [20] or lateral inclination of the trunk of ≥10° (Pisa syndrome) [21], (2) spinal disease or low back pain, (3) neurological disease other than PD, and (4) respiratory, circulatory, or metabolic disease.

All individuals in the PD group had been diagnosed by the same neurologist before their participation in the study. The doctor who examined them confirmed their modified H-Y Stage, duration of disease, and the side on which their PD symptoms first appeared. During the screening, they also confirmed that they could perform the Sørensen back endurance test in the correct position. All patients with PD performed measurements of maximum muscle strength during trunk extension and the Sørensen back endurance test during the on phase. The control group comprised nine healthy older women aged ≥65. All individuals in the control group were examined by a single rehabilitation doctor, who confirmed that they were in good health and that their participation in the study was appropriate.

### Isometric maximum voluntary contractions (MVC)

**Fig. 1:**
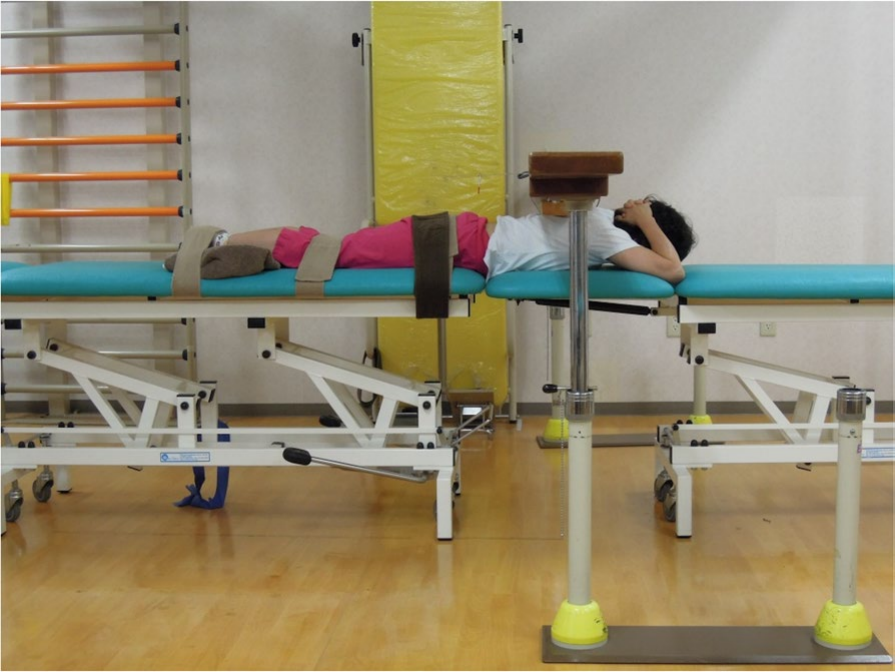
Body position during isometric maximum voluntary contraction measurements of trunk extension

After a 15-minute resting period, the subjects lay prone on two examination beds of adjustable height. Their legs were positioned so that their anterior superior iliac spines were aligned with the edge of one of the examination beds, and their hips, knees, and ankles were fixed to the bed with strong straps. The height of the other examination bed was adjusted so that it supported the trunk at 0° trunk extension. A handheld dynamometer (mTas F-1, ANIMA, Tokyo, Japan) was fixed at the midpoint of a line joining the inferior angles of the bilateral scapulae when both elbows were directed outwards, and both hands were placed on the back of the head (Fig. 1). Trunk extension isometric MVC was then measured three times for 3 s each time, separated by 30-s intervals of rest. To normalize the MVC, the torque values were expressed as Newton meters (Nm) and divided by body weight (Nm/kg). We have previously confirmed that this measurement method is highly reliable [13].

### Sørensen back endurance test

**Fig. 2:**
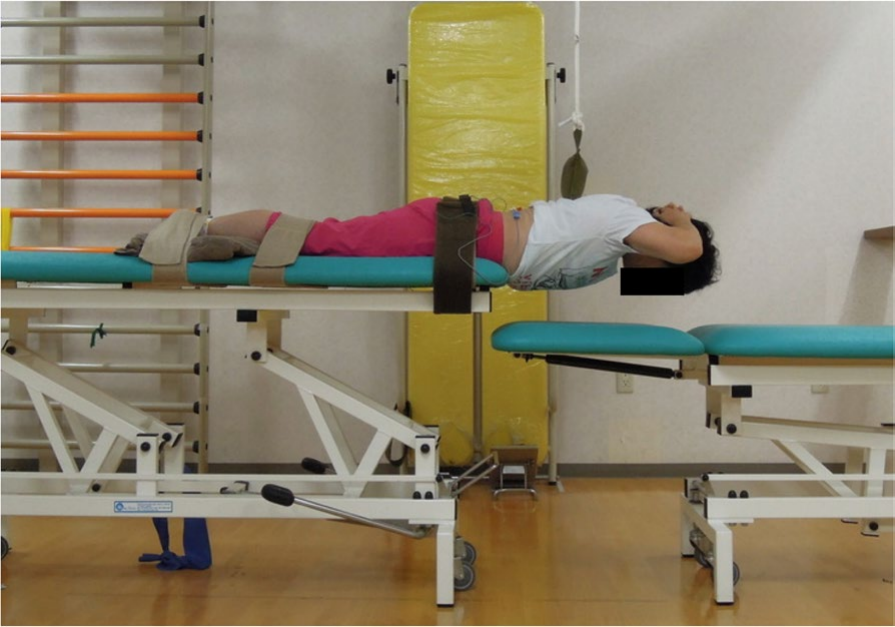
Body position during the Sørensen back endurance test

After the MVC measurements, the participants rested for 30 minutes to recover sufficiently from their fatigue. They then again laid prone on two examination tables of adjustable heights, with their legs positioned so that their anterior superior iliac spines were aligned with the edge of one of the examination beds, and their hips, knees, and ankles fixed to the bed with strong straps, in the same way as for the MVC measurements. The height of the other examination bed was adjusted so that it supported the trunk at 0° trunk extension. While both elbows were directed outwards and both hands placed on the back of the head, the height of the examination bed supporting the trunk was lowered while the subjects maintained the horizontal position of their trunk for as long as they could (Sørensen back endurance test: Fig. 2). For participants to be able to tell whether or not their trunk was still horizontal, a 0.5-kg weight was suspended from the ceiling so that it was at the height of the midpoint of a line joining the inferior angles of the bilateral scapulae when the trunk was at 0° extension, and the participants were instructed that this weight should not be lifted up but should lightly touch their back during the Sørensen back endurance test. The measurement was concluded when the trunk dropped more than 2 cm below the weight despite verbal encouragement, and the time from the start of measurement until this point was taken as the holding time.

### EMG signal recording and analysis

Erector spinae muscle activity during the Sørensen back endurance test was measured with an MQ 16 (Marq-Medical of Denmark, Farum, Denmark). The skin was wiped clean with alcohol before the electrodes were applied. Two silver–silver chloride electrodes (diameter 10 mm) were positioned 20 mm apart, with the leading electrode attached 3 cm laterally to the first lumbar vertebra so that it was parallel to the direction of the muscle fibers. Based on the Standards for Reporting EMG Data [22] approved by the International Society of Electrophysiology and Kinesiology, the recorded interference pattern was processed with an 8–500 Hz band pass filter, digitized with a Vital Recorder 2 analog-to-digital converter (Kissei Comtec, Nagano, Japan), and imported into a computer at a 2000-Hz sampling rate. The MF of the imported EMG signal was calculated at 1-s intervals by using the Kinema Tracer Fast Fourier Transform spectrum analysis program (Kissei Comtec), and the regression line was drawn to obtain the initial MF and MF slope.

### Statistical analysis

Data were expressed as medians and interquartile ranges (IQR). The proportions of participants in the PD and control groups who were able to perform the Sørensen back endurance test were compared using Fisher’s exact test. The physical characteristics of the patients with PD who were able and unable to perform the Sørensen back endurance test and those of the control group participants were compared using the Kruskal-Wallis test. PD-related parameters among patients with PD who were unable to perform the Sørensen back endurance test were compared using the Mann-Whitney U test. Because a number of previous studies have reported comparisons using the left erector spinae [11, 12, 14], the initial MF and MF slope of the control group were calculated from the left erector spinae as data for use in the MF comparisons. Comparisons between the initial MF and MF slope for the side on which PD symptoms first appeared and the opposite side, as well as with the control group, were conducted using the Dann-Bonferroni test following the Kruskal-Wallis test. Associations between two variables were investigated by using Spearman’s rank correlation coefficient. Statistical analysis was carried out using the Statistical Package for Social Sciences software, version 23.0, for Windows (SPSS Inc., Chicago, IL), with *p* < 0.05 considered significant.

## Results

### Proportion of patients able to perform the Sørensen back endurance test and participants’ physical characteristics

Of the 19 patients in the PD group, 10 were able to perform the Sørensen back endurance test, but the other nine were incapable of holding their trunk in the horizontal position from the very beginning. All nine members of the control group were able to perform the Sørensen back endurance test, and the difference in the proportion of participants in each group capable of performing this test was significantly lower in the PD group (*p* = 0.026). The patients with PD who were able to perform the Sørensen back endurance test, those who were unable to perform it, and the control group participants were similar in terms of age, height, weight, and body mass index. There was no significant difference between those who were able and unable to perform the Sørensen back endurance test in terms of either modified H-Y Stage or disease duration. The participants’ physical characteristics are shown in Table 1.

**Table 1:** Physical characteristics of the participants

	PD group			*p* value
	Capable of Sørensen test	Incapable of Sørensen test	Control group	
Age [years, median (IQR)]	70.0 (66.8-72.8)	74.0 (70.0-76.0)	70.0 (69.0-73.0)	0.37
Height [cm, median (IQR)]	156.3 (152.9-158.9)	150.7 (149.6-154.0)	149.0 (146.2-157.0)	0.09
Weight [kg, median (IQR)]	48.0 (40.6-54.0)	48.8 (45.0-53.9)	50.5 (45.0-51.4)	0.85
Body mass index [kg/m^2^, median (IQR)]	19.9 (17.3-22.2)	22.0 (20.0-22.7)	21.5 (20.7-24.0)	0.29
Modified H-Y Stage [median (IQR)]	1.8 (1.1-2.0)	1.0 (1.0-2.5)	N/A	0.60
Disease duration [months, median (IQR)]	44.5 (21.8-51.3)	16.0 (11.0-47.0)	N/A	0.40

*IQR* Interquartile, *H-Y* Hoehn-Yahr

### MVC and holding time

**Fig. 3:**
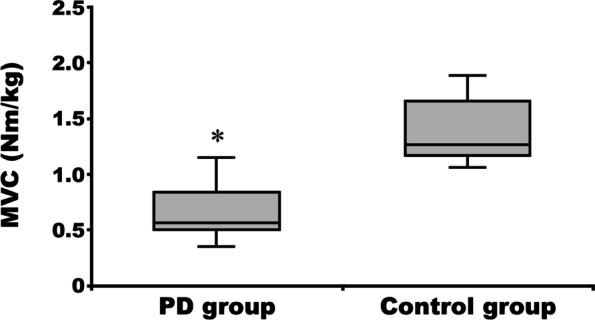
Maximum voluntary contractions (MVC) of the trunk extensor muscles in the Parkinson’s disease and the control group. *p < 0.01 vs control group

**Fig. 4:**
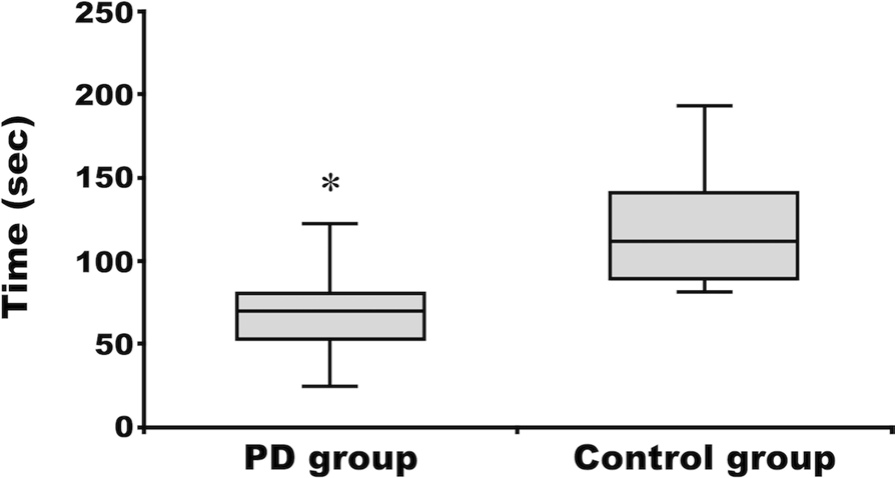
Holding time in the Parkinson’s disease and the control group. *p < 0.01 vs control group

The MVC values and Sørensen back endurance test results for the PD group are presented for the 10 patients who were able to perform the Sørensen back endurance test. Trunk extension isometric MVC was significantly lower in the PD than the control group (PD group: 0.57 (0.52–0.71) Nm/kg; control group: 1.26 (1.19–1.60) Nm/kg, *p* < 0.001) (Fig. 3). Holding time in the Sørensen back endurance test was significantly shorter in the PD than in the control group (PD group: 70 (54–76) s; control group: 112 (90–135) s: *p* = 0.001) (Fig. 4). There was no significant relationship between MVC and holding time (r = 0.578, *p* = 0.08). Neither MVC nor holding time was significantly associated with modified H-Y Stage or disease duration (*p* ≥ 0.05).

### MF index

**Fig. 5:**
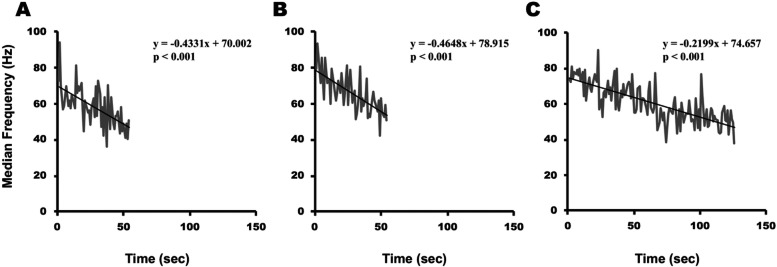
Changes over time in median frequency on the side on which the symptoms first appeared in a 76-year-old Parkinson’s disease patient (**A**), on the opposite side of the same patient (**B**), and on the left side of a 65-year-old healthy older woman (**C**)

**Fig. 6:**
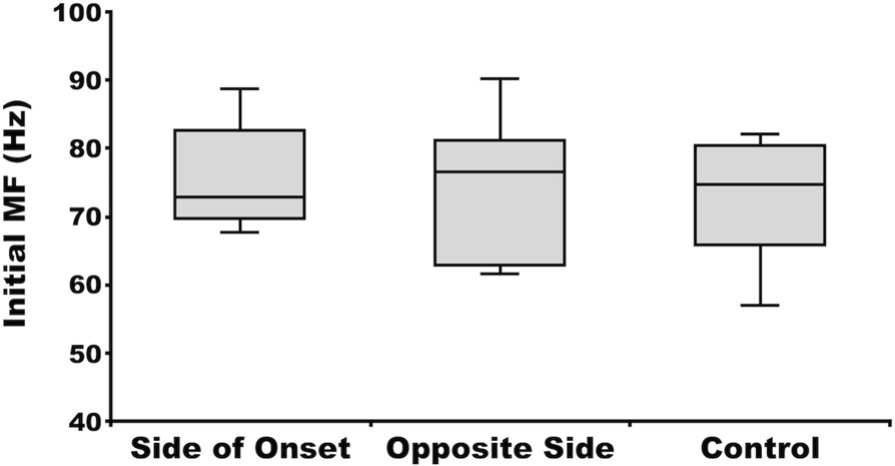
Initial median frequency (MF) on the side on which the symptoms first appeared in patients with Parkinson’s disease, on the opposite side in those patients, and on the left side of patients in the control group

**Fig. 7:**
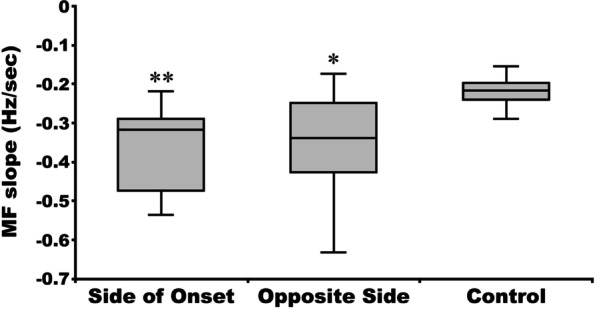
Median frequency (MF) slope on the side on which the symptoms first appeared in patients with Parkinson’s disease, on the opposite side in those patients, and on the left side of patients in the control group. *p < 0.05, **p < 0.01 vs control

The calculated MF values of the bilateral erector spinae attenuated significantly over time among both the 10 patients in the PD group and all nine control group participants (*p* < 0.001) (Fig. 5). There were no significant differences in the initial MF between the side on which symptoms first appeared in the PD group, the opposite side in the PD group, and the control group (*p* = 0.837) (Fig. 6). However, although there was no significant difference in the MF slope between the side on which symptoms first appeared and the opposite side in the PD group, both were significantly attenuated compared with the control group (Fig. 7). The MF slope on either the side on which symptoms first appeared or the opposite side in the PD group was not associated with MVC, holding time, modified H-Y Stage, or disease duration (*p* ≥ 0.05).

## Discussion

The main finding of this study is that only 53% of patients in the PD group were able to perform the Sørensen back endurance test, a significantly lower proportion than in the control group, which consisted of healthy individuals matched for age, sex, and physical characteristics (100%). The trunk extension MVC values of those patients in the PD group who were able to perform the Sørensen back endurance test were also significantly lower than those of the control group participants, and their Sørensen back endurance test holding times were significantly shorter. In addition, although there was no difference in the MF slope of the erector spinae between the side on which symptoms originally appeared and the opposite side in patients with PD, it was significantly attenuated in both compared with the erector spinae of healthy individuals.

Around half of the patients with PD in this study were unable to perform the Sørensen back endurance test. According to previous reports, the Sørensen back endurance test requires approximately 40% MVC [23]. The fact that around half of the patients with PD in this study were unable to perform the Sørensen back endurance test and that the mean MVC of those patients with PD who were able to perform it was equivalent to 50% of the mean MVC of the control group suggests that the majority of modified H-Y Stage < 3 patients with PD have a trunk extension muscle strength of less than 50% of that of healthy individuals of the same age. Some reviews [16, 24] have previously reported that the relative intensity of the Sørensen back endurance test varies by virtue of differences in age, body mass index, and muscle strength. In the current study, the PD group and the control group were matched for age, sex, and physical characteristics, but the possibility that the relative strength needed for the Sørensen back endurance test is higher because the extensor muscle strength of patients with PD is severely reduced must be kept in mind when interpreting the MF slope.

According to a previous study that combined histopathological testing and frequency power spectrum analysis [25], MF attenuation is more gradual when there is a higher proportion of type 1 muscle fibers, which have better endurance, and steeper when there is a higher proportion of type 2B muscle fibers, which lack endurance; the MF slope therefore provides an index of the fatigability of skeletal muscles. The erector spinae are postural muscles that contain predominantly type 1 muscle fibers [26], and the MF slope calculated from erector spinae activity during the Sørensen back endurance test is reportedly correlated with the proportion of type 1 fibers [10]. In the current study, the MF values calculated for the erector spinae of patients with PD exhibited similar attenuation on both sides, but this attenuation was steeper than that seen in the control group. That is, compared with the control group, the fatigability of the erector spinae in patients with PD was greater, suggesting that they may have contained a lower proportion of type 1 fibers than normal. However, previous studies have reported that the skeletal muscles of patients with PD exhibit fatigability despite their high type 1 fiber content [4], and it has been suggested that this may be due to mitochondrial dysfunction [5, 6]. We therefore conjecture that even if the proportion of type 1 fibers was high, a lack of mitochondrial activity may have reduced the efficiency of the aerobic metabolism, and the MF slope thus reflected the resulting quality of the erector spinae.

This study has a number of limitations. The first is the small sample size. However, because the patients with PD included in this study were all older women with a modified H-Y Stage < 3, and since we compared their results with those of healthy individuals matched for age, sex, and physical characteristics, we consider the clinical significance of our data high. The second limitation of the current study is that in our investigation of differences in MF slope between the PD and the control group, the severe reduction in erector spinae muscle strength in the patients with PD may have increased the relative load during the Sørensen back endurance test for the PD group. As this was a cross-sectional study, however, it is impossible to determine whether the difference in MF slope between the two groups was due to changes in the muscle fiber composition of the erector spinae or to differences in the relative intensity of the Sørensen back endurance test. In either case, however, this is the first study to demonstrate that the fatigability of the erector spinae, which contributes greatly to postural maintenance, is greater in patients with PD.

We found that only 53% of modified H-Y Stage < 3 patients with PD were able to perform the Sorensen back endurance test, and that trunk extensor muscle strength was greatly reduced and the fatigability of the erector spinae was higher in the PD group. However, there is no universal agreement with regard to the appropriate assessment of erector spinae muscle fatigability with the Sørensen back endurance test. Test subjects exhibited significantly longer holding times during the modified Sørensen back endurance test on a 45-degree Roman chair than on the original Sørensen back endurance test. Moreover, moderate and significant correlation between both tests was observed for holding time, and there was no difference in MF slope derived from erector spinae between both tests [27]. Therefore, the modified Sørensen back endurance test on a Roman chair might be a good choice to assess erector spinae muscle fatigability in patients with PD. However, erector spinae muscle fatigability data from the modified Sørensen back endurance test on a Roman chair is scant and the validity and reliability of the test therefore need to be more fully evaluated. In addition, previous studies reported that PD patients with postural abnormalities such as camptocormia, Pisa syndrome, and anterocollis have greater disability, higher risk of falls, and worse quality of life [28]. It is thus important to evaluate the correlation between erector spinae muscle fatigability and spine alignment, muscle volume, disability, fall tendency, and quality of life for patients with PD.

Our findings suggest that there is a marked decline in the quality of the erector spinae on both sides from the initial stage of PD, and that symmetrical strengthening of the endurance of the bilateral erector spinae from the early stage of the disease is required. An effective method of muscle strength training for the erector spinae in patients with PD has yet to be established. The trunk extension MVC of patients with PD is severely reduced, and we conjecture that high-intensity training may be clinically problematic in many cases. With respect to setting the intensity of muscle strengthening training, it has recently been suggested that low-intensity training to the point of exhaustion may activate both type 1 and type 2 fibers, leading to the same degree of muscle enlargement as high-intensity training [29]. As soon as they are diagnosed, patients with PD must therefore engage in bilaterally symmetrical muscle strengthening training of the erector spinae at a load that enables them to continue for as long as possible, under the medical management of a rehabilitation therapist or similar professional. Trunk extension exercises on a Roman chair would allow patients to train the erector spinae muscles more specifically by overloading them over a longer duration [30]. Aerobic exercise in a format that activates the muscle activity of the paraspinal muscles increases the aerobic metabolism, which contributes to increasing the size of type 1 muscle fibers [31] and may prevent the progression of postural abnormality. Regular rowing exercise has also been suggested to strengthen the erector spinae [32].

## Conclusion

To our knowledge, this is the first study to use surface electromyography power spectrum analysis to investigate the fatigability of the erector spinae in patients with PD. We found that only 53% of modified H-Y Stage < 3 patients with PD were able to perform the Sørensen back endurance test and that trunk extensor muscle strength was greatly reduced and the fatigability of the erector spinae was high in the PD group. Our findings strongly support the necessity of paraspinal muscle training at an early stage to prevent postural abnormality in patients with PD.

## Data Availability

All relevant data are presented within the manuscript. The dataset generated and analyzed in the current study is not publicly available due to privacy constraints relating to the ethical approval and informed consent signed by the participants. Data sharing was not stated in the informed consent signed by the participants.

## Abbreviations

PD: Parkinson’s disease
MVC: Maximum voluntary contraction
MF: Median frequency
MRI: Magnetic resonance imaging
EMG: Electromyography
H-Y: Hoehn & Yahr
